# Differential Diagnosis and Treatment of Disease

**Published:** 1906-02

**Authors:** 


DIFFERENTIAL DIAGNOSIS AND TREATMENT OF DISEASE. By Augustus Caillé, M.D. D. Appleton & Co., New York.

This book combines two of the most important features of mediicine, *i. e.*, the diagnosis and the treatment, and keeping them in the foreground covers all the diseases in the broad domain of medicine.

Special chapters are given on the Technique of Diagnosis and the

Laboratory Aids, on Pediatrics, and the various specialties, on Diseases of the Osseous, Muscular and Articular System, on Nutrition and Diet, on the management of Dropsy and Effusion, on Message, Vibration, Dry Hot Air Treatment, Poisons and Anesthesia.

From the standpoint of the general practitioner and the student this may be justly considered one of our best books on the practice of medicine.

